# Multiple Faces of Chronic Lymphocytic Leukaemia: A Patient with Renal, Cardiac, and Skeletal Complications

**DOI:** 10.1155/2019/5390235

**Published:** 2019-03-12

**Authors:** Zay Myo Htet, Cesar Gomez, Ahsan Ali, Sunil Nair, Mahzuz Karim

**Affiliations:** ^1^Department of Renal Medicine, Norfolk and Norwich University Hospital, Norwich, UK; ^2^Department of Haematology, Norfolk and Norwich University Hospital, Norwich, UK; ^3^Department of Cellular Pathology, Norfolk and Norwich University Hospital, Norwich, UK; ^4^Department of Cardiology, Norfolk and Norwich University Hospital, Norwich, UK

## Abstract

We describe a patient who had chronic lymphocytic leukaemia (CLL) Binet stage A at presentation with further evidence of disease at multiple sites but who initially required no treatment. However, several years later, her peripheral blood lymphocyte count started to increase, and soon after that she suffered an acute myocardial infarct (in the absence of coronary atheroma) together with proteinuric renal failure due to membranoproliferative glomerulonephritis. Her renal function improved markedly following anti-CLL chemotherapy. We postulate that her cardiac and renal disease were both complications of her CLL. In patients with CLL who develop new clinical signs or symptoms (even if apparently unrelated), consideration should be given as to whether these may be disease complications as this may serve as an indication to commence anti-CLL therapy; close liaison between different specialties is vital.

## 1. Case History

**Figure 1:**
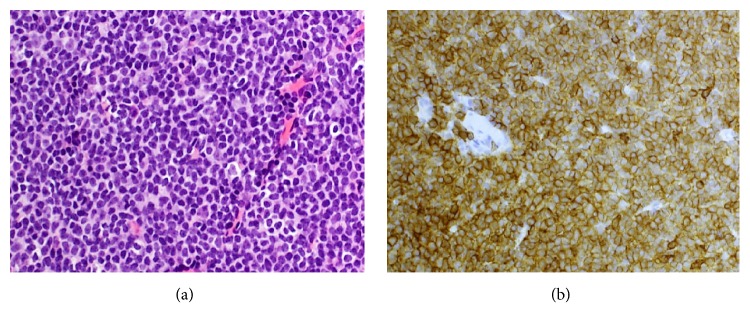
Axillary lymph node: (a) Haematoxylin and eosin stain showing monomorphic proliferation of small lymphocytes in keeping with CLL. (b) Immunoperoxidase stain showing positive staining of lymphocytes for CD5.

A 55-year-old woman was referred to the renal clinic in February 2016 with heavy proteinuria and deteriorating renal function. She had had a diagnosis of chronic lymphocytic leukaemia (CLL) for 6 years. This was first detected in August 2010 when a screening mammogram revealed right axillary lymphadenopathy. Core biopsy (Figure 1) demonstrated abnormal lymphoid tissue with small- to medium-sized lymphocytes with irregular nuclei. These cells stained positively for CD5, CD20, and CD23 but were negative for CD10 and cyclin D1. The proliferation index (Ki-67) was 5-10%. She had a staging computed tomography scan with contrast: this showed multiple enlarged cervical, axillary, and inguinal lymph nodes, but no mediastinal, abdominal, or pelvic lymphadenopathy and no hepatomegaly or splenomegaly. As she was asymptomatic and her full blood count was normal (including lymphocyte count 2.3 × 10^9^/L, reference range 1-3 × 10^9^/L), she required no active treatment but was kept under review.

**Figure 2:**
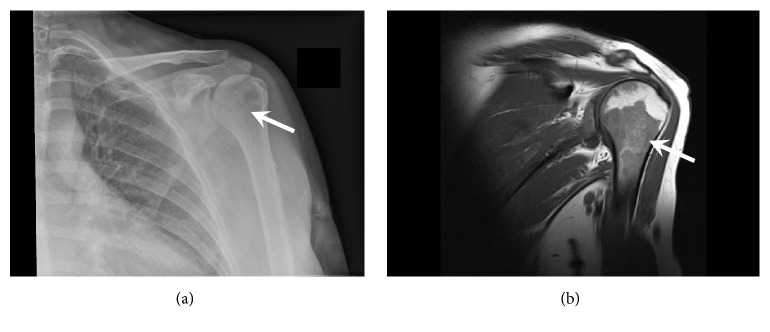
Imaging of left shoulder: (a) Plain radiograph showing lucency of humeral head. (b) MRI scan showing marrow infiltration of humeral head and shaft.

**Figure 3:**
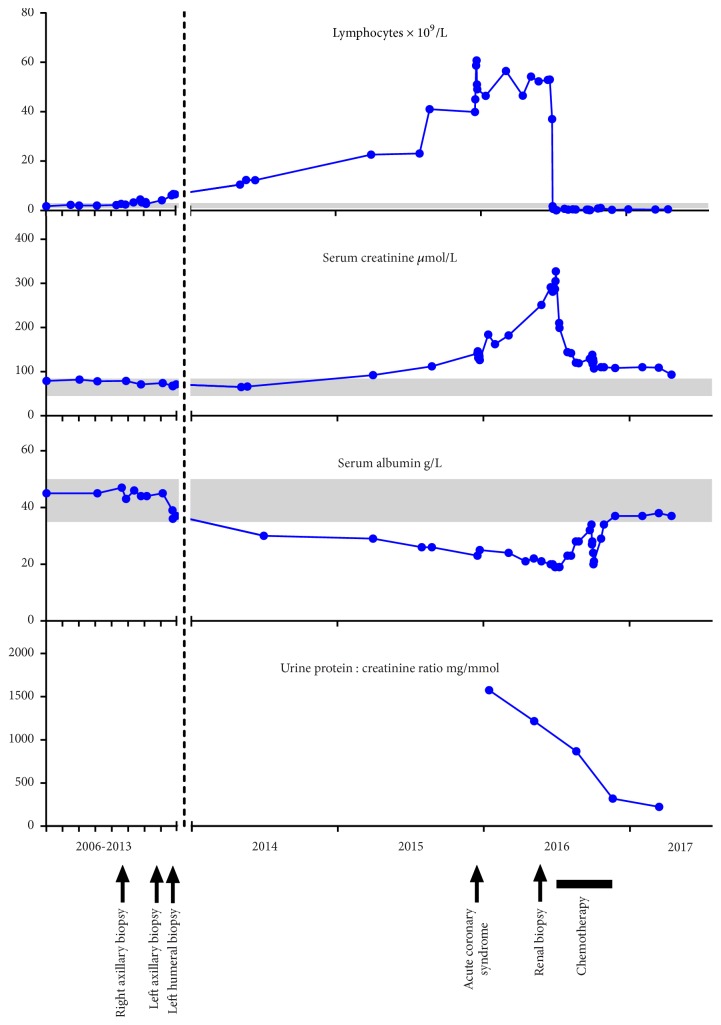
Patient's laboratory investigations and clinical events. Shaded areas represent reference ranges.

Two years later (April 2012), she developed a right breast mass. Excision biopsy showed an intraductal papilloma. A few months later, in November 2012, she noted some lumps in her left axilla; core biopsy was performed, and this was again consistent with CLL. Management remained conservative. In July 2013, she presented to the orthopaedic surgeons with a painful left shoulder. Plain radiography showed an ill-defined lucency projected over the lateral aspect of the left humeral head (Figure 2(a)), and magnetic resonance imaging (MRI, Figure 2(b)) revealed extensive marrow infiltration of the humeral head and shaft, shoulder girdle, and ribs, together with axillary lymphadenopathy. Bone biopsy confirmed infiltration by CLL but no evidence of high-grade transformation. Her peripheral blood lymphocyte count remained normal (6.57 × 10^9^/L). A serum IgG *λ* paraprotein was detected and quantified at 1.8g/L. During 2014, her lymphocyte count rose, and by early 2015 it was 22.6 × 10^9^/L (Figure 3).

**Figure 4:**
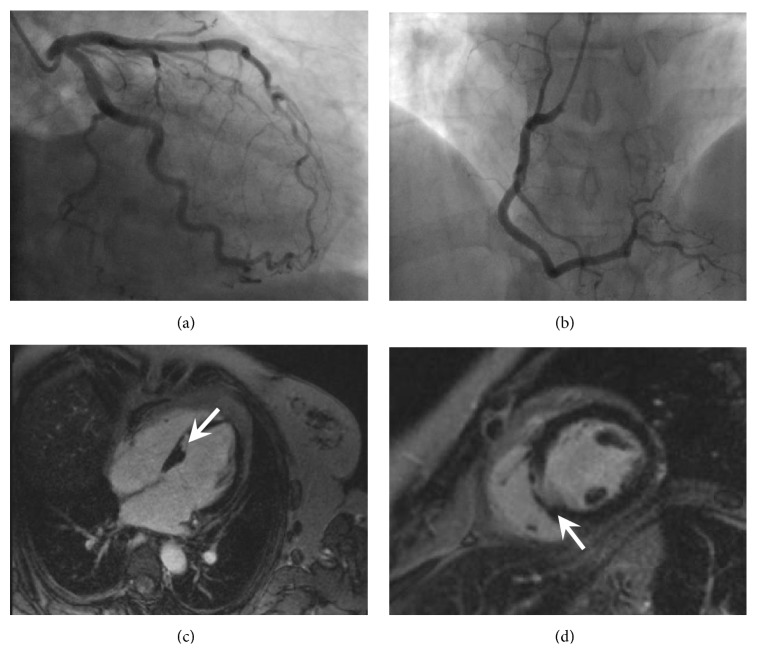
Cardiac imaging: (a) Coronary angiogram showing normal unobstructed left coronary artery. (b) Coronary angiogram showing normal unobstructed right coronary artery. (c) Left ventricle 4 chamber cardiac MRI late gadolinium enhancement image showing area of near transmural infarction in the mid septum. (d) Left ventricle short axis cardiac MRI late gadolinium enhancement image at the papillary muscle level showing area of near transmural infarction of the mid septum.

In November 2015, she presented with acute onset ischaemic chest pain. Although her ECG showed no acute changes, troponin I level was 44.5ng/L (0-15.6), climbing to 350.5ng/L two hours later; her lymphocyte count had also continued to rise (39.9 × 10^9^/L). Coronary angiography showed no significant stenosis or thrombus within the coronary arteries (Figures 4(a) and 4(b)). Antiplatelet therapy was commenced. Cardiac MRI demonstrated full-thickness enhancement in the midseptum with associated hypokinesia, consistent with an infarct (Figures 4(c) and 4(d)); left ventricular ejection fraction was well maintained at 72%. She underwent echocardiography to investigate the possibility of a paradoxical embolus as the cause for this infarct; this confirmed an area of hypokinesis in the mid-interventricular septum, but with no evidence of an atrial septal defect or patent foramen ovale. She had no further cardiac symptoms.

**Figure 5:**
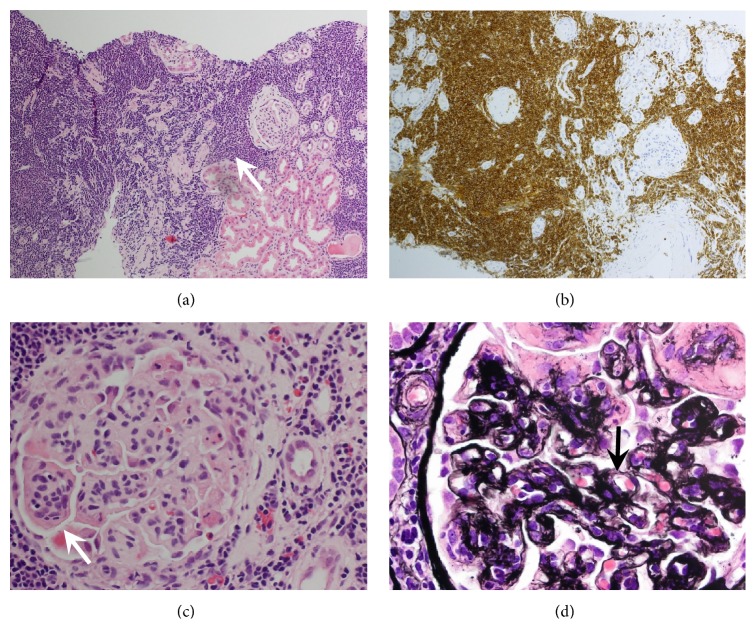
Percutaneous renal biopsy: (a) Haematoxylin and eosin stain showing dense lymphoid infiltrate. (b) Immunoperoxidase stain showing positive staining of lymphocytes for CD5. (c) Haematoxylin and eosin stain showing lobulation of glomeruli in keeping with MPGN. (d) Silver stain showing thickening and double contouring of basement membrane in keeping with MPGN.

Referral to the renal clinic in February 2016 was prompted by a decline in renal function (Figure 3). Her serum creatinine had risen progressively from 66 *μ*mol/L (45-84) two years earlier (April 2014) to 182 *μ*mol/L. Lymphocyte count was 56.5 × 10^9^/L, without anaemia or thrombocytopaenia. Serum albumin was low at 24 g/L (35-50). Urine dipstick testing showed 3+ blood and 4+ protein, with microscopy showing red cells but no casts; urine protein:creatinine ratio was elevated at 1574 mg/mmol (0-20). Her serum IgG *λ* paraprotein level remained low at 1.0g/L, with serum free *λ* chains 96.5mg/L (5.7-26.3), serum free *κ* chains 24.4mg/L (3.3-19.4), and *κ*:*λ* ratio 0.25 (0.26-1.65). Other normal investigations included anti-nuclear antibodies 0.1 U/L (0-1), negative anti-neutrophil cytoplasmic antibodies by immunofluorescence, proteinase 3 <0.1 U/mL (0-3), myeloperoxidase <0.1 U/mL (0-5), complement C3 1.4g/L (0.75-1.65), C4 0.27g/L (0.14-0.54), rheumatoid factor <20 U/mL (<30), negative cryoglobulins, and negative hepatitis B and C serology. She underwent percutaneous renal biopsy (Figure 5). This showed an interstitial lymphocytic infiltrate (CD20 and CD5 positive), consistent with CLL. Additionally, the glomeruli showed membranoproliferative change with thickening and double-contouring of the basement membrane and immunostaining positive for IgG and C3 but negative for IgA and IgM; staining for *κ* and *λ* light chains was not performed. Congo red and amyloid P stains were also negative. These changes were felt secondary to her CLL, and she was therefore commenced on treatment with six cycles of rituximab and bendamustine delivered every four weeks between June and November 2016. Clinical response was excellent, with her serum creatinine falling from a peak of 327 *μ*mol/L to 120 *μ*mol/L within six weeks of starting treatment. Computed tomography scan in December 2016 showed no evidence of lymphadenopathy, and in February 2017, three months after completing therapy, serum creatinine was 109 *μ*mol/L, albumin 38 g/L, and lymphocyte count 0.4 × 10^9^/L; however, she still had significant proteinuria with a urine protein:creatinine ratio of 226 mg/mmol. Serum electrophoresis revealed only a faint IgG *λ* paraprotein band which was too small to quantify. Echocardiogram showed a left ventricular ejection fraction of 63%, with no regional wall motion abnormality.

## 2. Discussion

CLL is one of the commonest haematological malignancies in adults, with an incidence of around 600 per million in the Western world [1]. Clinical presentation is variable and may include features such as asymptomatic peripheral lymphocytosis, lymphadenopathy, increased incidence of infection, autoimmune phenomena (e.g., haemolytic anaemia, thrombocytopaenia), and B symptoms (fever, unintentional weight loss, night sweats, and severe fatigue) [1].

The clinical course of CLL can vary greatly. Many patients remain asymptomatic for decades and require no specific intervention. However, factors that may mandate active treatment include disease stage, evidence of disease progression (e.g., cytopaenias, progressive lymphadenopathy, and/or splenomegaly), and the presence of constitutional B symptoms, or when transformation to high-grade lymphoma occurs (Richter's transformation) [1]. Our patient had CLL with deposits at multiple sites (including lymph nodes, breast, and bone) but initially required no treatment as she was otherwise asymptomatic. However, soon after her peripheral blood lymphocyte count began to rise, she developed an acute coronary syndrome followed by heavy proteinuria and worsening renal function.

Renal insufficiency is not uncommon in patients with CLL, present in one study in 7.5% of patients at diagnosis, rising to 16.1% during follow-up [2]. Lymphocytic infiltration of the kidneys (that can mimic an acute or chronic interstitial nephritis) is frequent, occurring in up to 90% of patients at autopsy, but does not have a significant impact on renal function in the majority of cases [3]. Other potential causes of renal dysfunction include volume depletion, sepsis, nephrotoxic drugs, hypercalcaemia, tumour lysis syndrome, thrombotic microangiopathy (TMA), and obstructive uropathy (e.g., ureteric compression by lymphadenopathy) [4]. More rarely, there can be an associated glomerulonephritis (GN) as in our patient.

In two large retrospective studies of renal biopsies in patients with CLL and small lymphocytic lymphoma (SLL), membranoproliferative glomerulonephritis (MPGN, also known as mesangiocapillary GN) was the commonest lesion; others included minimal change disease, membranous GN, light chain cast nephropathy, TMA, and amyloidosis [4, 5]. The pathophysiology of MPGN in CLL is thought to involve deposition of paraprotein M components [6], for example, cryoglobulinaemia (most commonly type 2) with activation of the complement system, or noncryoprecipitating monoclonal IgG deposition without complement activation [7]. This has been termed “proliferative glomerulonephritis with monoclonal IgG deposits” (PGNMID) and can be mistaken as an immune-complex GN due to the fact that they both share similar histopathological findings (membranoproliferative or endocapillary proliferative GN with nonorganized granular deposits) [8]. More recently, it has been recognised that monoclonal IgM deposition may also lead to MPGN (although only two cases have been reported in association with CLL) [9, 10], described as “proliferative glomerulonephritis with monoclonal IgM deposits” (PGNMIMD) [9–11].

The clinical presentation of MPGN is most frequently a nephritic picture with features such as hypertension, haematuria, proteinuria (often nephrotic range), active urine sediment, and renal impairment. The next commonest indication for renal biopsy in CLL is nephrotic syndrome [4, 5, 12]. Since the signs and symptoms of renal involvement may be mild or subtle, even with fairly advanced disease, it is crucial to maintain a high index of suspicion and a low threshold for renal biopsy. Additionally, renal insufficiency has been shown to be an independent prognostic indicator in CLL and can influence therapeutic decisions [13]. Renal manifestations can regress following treatment of the underlying CLL [4, 5], and thus where renal involvement is identified, initiation of appropriate anti-CLL therapy should be considered. In some cases, the decision to initiate treatment may not be straightforward, and renal dysfunction can restrict the choice of chemotherapeutic agent(s). Our patient showed an excellent clinical improvement after six cycles of rituximab and bendamustine. To our knowledge, this is the third report of a case of CLL-associated proliferative GN managed successfully with a rituximab and bendamustine-based regimen leading to substantial renal improvement [14].

The other interesting aspect in this patient is her acute coronary syndrome. She had typical ischaemic symptoms and a troponin rise with MRI and echocardiogram showing full thickness infarction and hypokinesia, but in the absence of a prior cardiac history or traditional cardiovascular risk factors and with normal coronary angiography. As this happened at a time when her disease was progressing (demonstrated by a rising peripheral lymphocyte count and worsening renal function), we hypothesize that her CLL played a pathophysiological role.

Although cardiac infiltration by CLL and other lymphoproliferative disorders is well-described (occurring in 20% of patients in one postmortem series) [15], reports of consequent cardiac dysfunction are less common. A high peripheral leucocyte count itself has been demonstrated to increase the risk of (and mortality from) acute coronary artery thrombosis and to stimulate chronic atherosclerosis and microvascular occlusion. Multiple potential mechanisms have been suggested including leucocyte aggregation and adhesion with vessel plugging, activation of the coagulation system, interaction with platelets to promote thrombin generation, increased expression of monocyte and neutrophil tissue factors, and release of soluble mediators such as proteases, reactive oxygen species, growth factors, interleukins, and myeloperoxidase [16–18]. Patients with CLL may also develop amyloid infiltration of the heart, either paraprotein-related AL amyloid [19] or, less commonly, non-AL; the latter group appears to have a better prognosis [20]. Amyloid can also deposit within vessels causing occlusion [21]. CLL infiltration of the pericardium may lead to constrictive pericarditis [15, 22] and of the aortic valve to aortic stenosis [23].

In 1980, Applefield* et al*. reported a 47-year-old man with CLL who died from congestive cardiac failure. Postmortem examination showed endocardial fibroelastosis and leukaemic infiltration of the endocardium, myocardium, and coronary arteries [24]. More recently, Assiri* et al*. described an 83-year-old man with CLL who died following an acute myocardial infarct. Autopsy showed infiltration of the coronary artery walls by leukaemic cells but, unlike our patient, also significant atheromatous disease [25]. Finally, Xu* et al*. documented a 65-year-old woman with Richter's progression of CLL who presented with an acute myocardial infarct. She had a peripheral leucocyte count of 316 × 10^9^/L and an IgM paraprotein of 0.3g/dL. She underwent plasmapheresis to reduce her leucocyte count but died despite this. Postmortem examination showed lymphomatous infiltrates in the myocardium, epicardium, and endocardium with occlusion of small vessels; immunohistochemistry showed IgM heavy chain and *λ* light chain deposition in a subintimal distribution [26]. It is thus plausible that our patient's acute cardiac event was related to her CLL.

In summary, new clinical signs or symptoms in patients with CLL should prompt consideration of whether these may represent disease complications and thus be an indication to commence treatment for CLL, even in the absence of more classical criteria. Close liaison between different medical specialties is vital in optimising outcome.
